# Decreased Hippocampal 5-HT and DA Levels Following Sub-Chronic Exposure to Noise Stress: Impairment in both Spatial and Recognition Memory in Male Rats

**DOI:** 10.3797/scipharm.1207-15

**Published:** 2012-10-07

**Authors:** Saida Haider, Fizza Naqvi, Zehra Batool, Saiqa Tabassum, Tahira Perveen, Sadia Saleem, Darakhshan Jabeen Haleem

**Affiliations:** 1 Neurochemistry and Biochemical Neuropharmacology Research Unit, Department of Biochemistry, University of Karachi, Karachi-75270, Pakistan.; 2 Neuroscience Research Laboratory, Dr. Panjwani Center for Molecular Medicine and Drug Research, University of Karachi, Pakistan.

**Keywords:** Spatial memory, Recognition memory, Noise, Serotonin, Dopamine, Hippocampus

## Abstract

Mankind is exposed to a number of stressors, and among them noise is one which can cause intense stress. High levels of background noise can severely impair one’s ability to concentrate. The present study was aimed to investigate the effect of sub-chronic noise stress on cognitive behavior and hippocampal monoamine levels in male rats. The study was performed on 12 male Wistar rats, divided into two groups; the control and noise-exposed. The rats in the test group were subjected to noise stress, 4h daily for 15 days. Cognitive testing was performed by the Elevated Plus Maze test (EPM) and Novel Object Recognition test (NOR). HPLC-EC was used to determine hippocampal monoamine levels and their metabolites. The data obtained revealed a significant decrease in hippocampal serotonin (5-hydroxytryptamine; 5-HT) and dopamine (DA) levels, whereas turnover ratios of 5-HT and DA were significantly increased compared to the controls. Rats exposed to noise exhibited a significant decrement in spatial memory. A significantly decreased recognition index of rats exposed to noise as compared to the control was also observed in the NOR test. Results of the present findings suggest the role of decreased hippocampal 5-HT and DA in the impairment of cognitive function following noise exposure.

## Introduction

Stress is an unavoidable phenomenon that affects the body systems at various levels [1]. Among the most widespread environmental stressors, noise is the highest encountered stressor that effects living beings throughout the world [1, 2]. It is a widely accepted fact that noise is a stressful stimulus [1] that induces stress in both animals and humans and disrupts the activity or balance of life [3]. As a stressor, noise has a great influence on the human body [3]. Living in a noisy environment not only causes psychological, physiological, or behavioral changes in people, but also affects sleep, work efficiency, performance, and communication ability [3, 4]. The brain, the key organ that interprets and responds to potential stressors [5], recognizes the sound levels and discriminates the stress levels. It reacts within split seconds to help the body adjust to this stressful situation by releasing hormones [2]. Researchers demonstrated that noise exposure is a potent stressor that increases the levels of the stress hormone, corticosterone [6]. Noise stress effects are not sudden and catastrophic but gradual and insidious [2]. Interactions between noise stress-inducing mechanisms and physiological systems involved in combating noise may lead to noise-induced illness [2].

Reports have shown that noise exposure affects mental health and performance and induces behavioral changes [7]. Earlier studies have documented that exposure to occupational noise adversely affects the cognitive task performance of workers and impairs the number of cognitive and motivational parameters of children [7–9]. Among the cognitive effects, work, attention, problem solving, and memory are most strongly affected by noise [9]. A study on animals showed that exposure to noise caused the impairment of spatial memory [10]. The brain handles repeated stress by showing adaptive plasticity that produces structural as well as functional changes in systemic hormones and local neurotransmitters [2, 5]. Reports have shown that noise exposure alters the biogenic amine levels in the brain [1, 11]. DA and 5-HT are implicated in environmentally-induced behavioral disorders [12], whereas evidence also shows the role of 5-HT and DA in cognition, memory function [13, 14], and other behaviors [15]. Spatial learning and memory are specifically associated with the hippocampus [7]. Studies show that exposure to noise stress impairs neurogenesis in the rat hippocampus and may affect neurotransmission in this region [16]. Since there seems to be a relationship between noise, hippocampal damage, and memory impairment, this study was therefore designed to monitor the effects of noise stress on hippocampal 5-HT, DA, and cognitive behavior in rats.

## Results

**Fig. 1.**
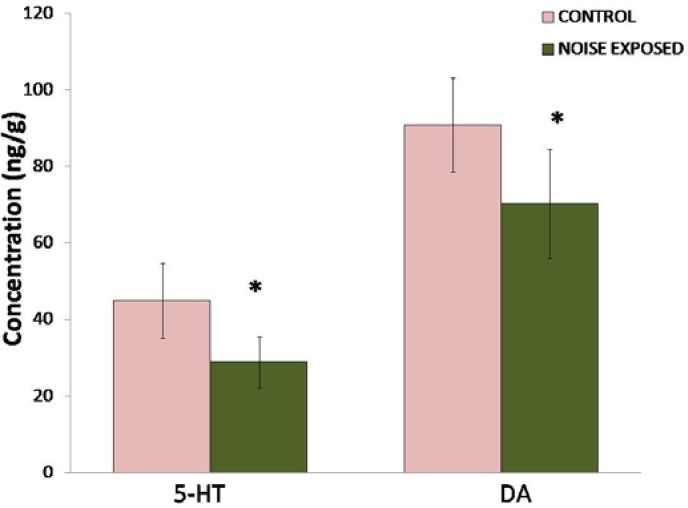
Effect of noise stress on the levels of hippocampal 5-HT and DA. Values are presented as mean ± SD (n=6) and significant differences by Student’s t-test are shown as * P<0.05 with respect to controls.

**Fig. 2.**
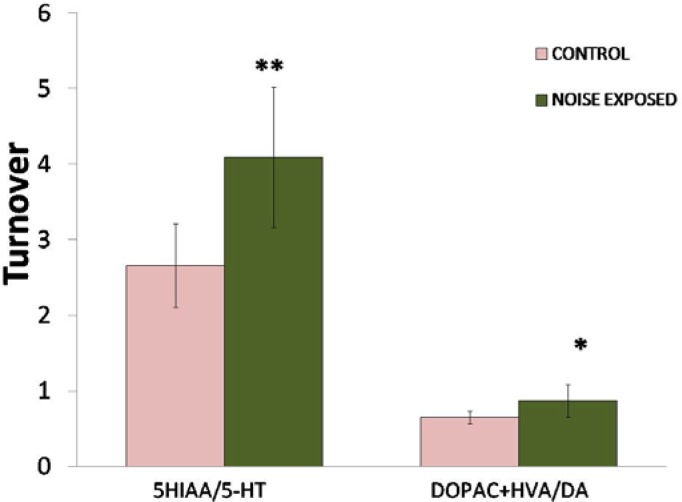
Effect of noise stress on the levels of 5-HT and DA turnover in the hippocampus of rats. Values are presented as mean ± SD (n=6) and significant differences by Student’s *t*-test are shown as * P<0.05, ** P<0.01 with respect to controls.

A significant decrease (P<0.01) in both hippocampal 5-HT and DA levels was observed in rats exposed to noise compared to controls (Fig. 1). Noise stress caused a 35.7% decrease in 5-HT and 22.7% decrease in DA. However, turnover of serotonin (P<0.01) and dopamine (P<0.05) was significantly increased (Fig. 2).

**Fig. 3.**
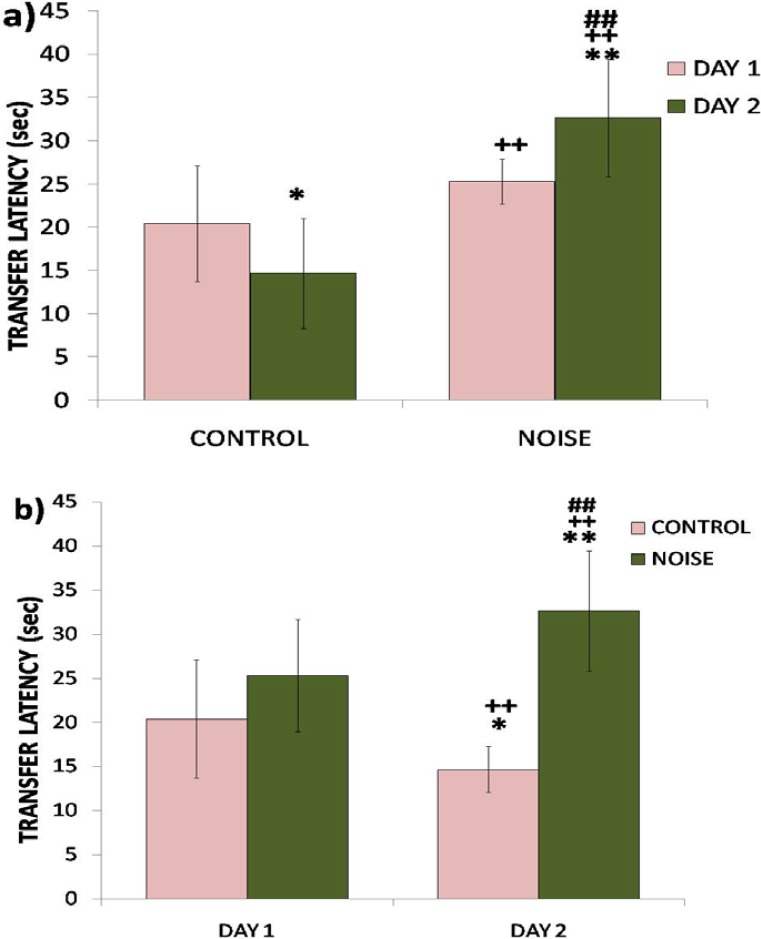
Effect of noise stress on spatial memory performance of rats assessed by EPM in terms of transfer latency during subsequent phases of learning (a) and retention (b). Values are presented as mean ± SD (n=6) and significant differences by two-way ANOVA are shown as * P<0.05, ** P<0.01 with respect to controls.

The transfer latency on the first day reflected impairment in learning (P<0.05), whereas the transfer latency on the 2nd day reflected impairment in retention (P<0.01) of the learned task (Fig. 3a). The rats exposed to noise showed a significant impairment in spatial memory (P<0.05). However, control rats exhibited a non-significant improvement in memory as shown by a decrease in transfer latency on day two (Fig. 3b).

**Fig. 4.**
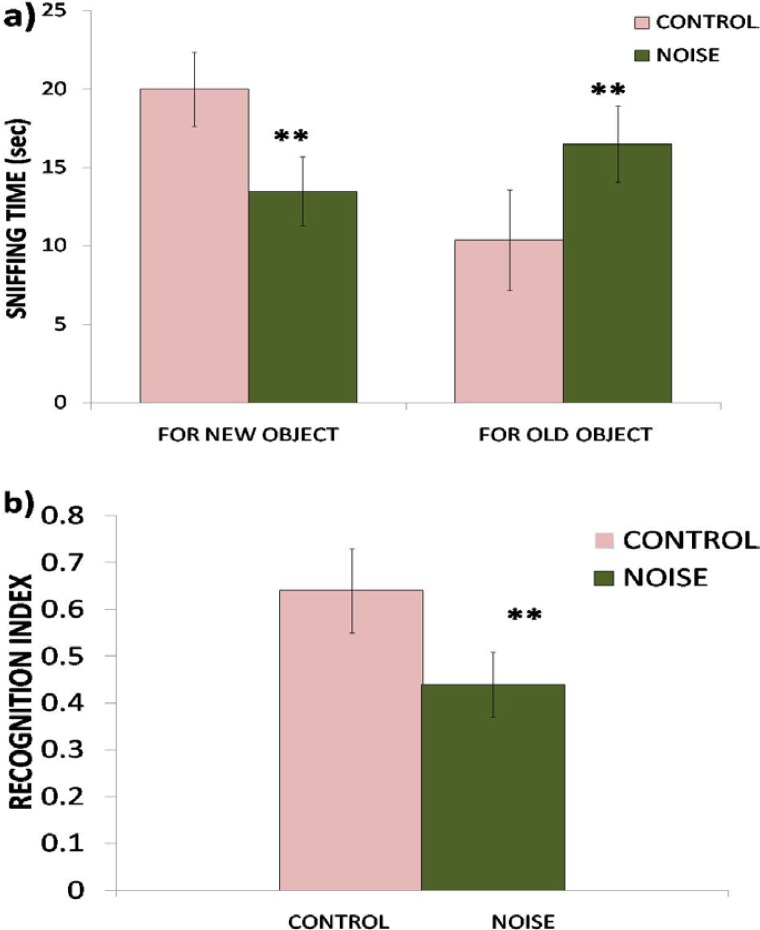
Effect of noise stress on recognition memory of rats assessed by NOR in terms of sniffing time (a) and recognition index (b). Values are presented as mean ± SD (n=6) and significant differences by Student’s *t*-test are shown as ** P<0.01 with respect to controls.

In the recognition test, rats exposed to noise showed a significant (P<0.01) decrease in the time spent exploring the novel object (Fig. 4a) and therefore exhibited impairment in recognition memory revealed by a significantly (P<0.01) decreased recognition index compared to controls (Fig. 4b).

## Discussion

Noise, being one of the most widespread sources of environmental stress, has been shown to produce a number of physiological, biochemical, and neurochemical responses in both human and animals. Both long-term as well as acute exposure to noise effects the central nervous system.

The overall results of our study showed that noise exposure for 15 days altered the levels of serotonin and dopamine in the hippocampus and produced working memory and recognition memory deficits in rats. The adverse effects of noise exposure observed on spatial and recognition memory are in agreement with the findings of other researchers [7, 17, 18]. In the present study, the performance of rats exposed to noise was significantly impaired in both memory tasks. In the EPM test, the increase in the transfer latency of the rats exposed to noise compared to controls on the first day reflected impairment in learning (P<0.05), whereas an increase in transfer latency of noise-exposed rats on the 2^nd^ day reflected impairment in retention (P<0.01) of the learned task. A decrease in transfer latency of control rats on the 2^nd^ day was statistically insignificant, however, when compared with the respective first day values, which indicates a tendency of improvement in the performance of rats not exposed to noise stress. It is widely accepted that noise is a stressful environmental stimulus and stress has been previously shown to impair cognition such as the acquisition of memory, consolidation, and recall [19, 20]. Exposure to noise stress in the present study also impaired recognition memory in the NOR test. The rats in the noise-exposed group showed a significant (P<0.01) decrease in time spent exploring the novel object and hence exhibited a marked impairment in recognition memory.

Neurobiological studies on cognition show that the hippocampus is the key region critically involved in memory formation [21] and a primary target of stress hormones [22]. Furthermore, stress has also been shown to suppress neurogenesis [23]. Although corticosterone levels were not determined in the present study, evidence exists showing increased corticosterone levels following noise exposure [2, 18]. The impaired memory function exhibited by noise-exposed rats in the present study may be attributed to noise stress-induced hippocampal damage [23]. Increased plasma glucocorticoids have previously been shown to effect neurogenesis [23]. Monoamine neurotransmitters play a key role in memory function, and changes in hippocampal monoamine levels have been implicated in altered learning and memory performance [24]. In Revindran’s study, rats were exposed to noise for 15 days which was found to increase biogenic amines in discrete brain regions. In another study, rats exposed to noise for 30 days exhibited a decrease in brain biogenic amines [17]. In our study, 15-day exposure to noise decreased 5-HT and dopamine levels while increasing their turnover ratios in the hippocampus. The decreased 5-HT and dopamine levels could be the result of the deleterious effects of noise on the hippocampus [16, 23]. The stress-induced stimulation of serotonergic and dopaminergic systems has been regarded as plasticity that helps the brain reorganize its neuronal network [25]. The decrease in hippocampal 5-HT and DA levels in noise-exposed rats may therefore influence the plasticity of synapses resulting in impairment of memory as observed in this study. Both 5-HT and DA have a major role in learning and memory functions [13, 14, 24, 26]. Previously, it has been shown that administration of the 5-HT precursor tryptophan increased 5-HT levels more in the hippocampus than in any other region of the brain and also enhanced the memory function in rats [13, 27]. On the other hand, decreased brain tryptophan and 5-HT levels have been shown to impair memory function [28]. The role of the dopaminergic system in many behavioral and biological functions of the CNS is also well established [29]. Altered dopaminergic transmission has been implicated in cognitive impairment [14].

## Conclusions

In the present study, we report that 15-day exposure to noise stress induces working and recognition memory deficits in rats. This impairment in memory function may be linked to the altered 5-HT and DA levels observed in the hippocampus of rats exposed to noise stress. Further studies regarding changes in neurotransmitter receptors and the glucocorticoid receptor following exposure to noise would be useful to better understand the mechanism involved in noise-induced behavioral deficits and help design strategies to combat the harmful effects of noise.

## Experimental

### Animals

The experimental animals were all healthy adult male albino rats of the Wistar strain weighing 180–250g. The animals were purchased from the animal house of the HEJ Institute of Chemistry and were used in the study. All animals were housed individually under a 12h light-dark cycle (light on at 6:00 h) and controlled room temperature (22+2°C) with free access to cubes of standard rodent diet and tap water for at least 3–4 days before experimentation so that the rats could adapt to the new environment.

### Experimental Design

The animals were randomly divided into the control and noise-exposed groups. The animals of the test group were exposed to noise for 4h (9:00 am – 1:00 pm) daily for 15 days. Noise was recorded from the generator and amplified by speakers in a separate room. Speakers were located 30cm above the cages. The noise level was set at 100 dB and intensity was measured by a sound level meter DS102 (Range: 80–130 dB, Accuracy: +1.5 dB, made in Taiwan). The control group of rats was kept in the same room for the same period of time without switching on the noise. 24 hrs after the last noise stress session, behavioral analysis was done to assess the spatial and recognition memory by EPM & NOR respectively, with the time interval of about 24 hrs. One hr after behavioral analysis, rats were decapitated between 10.00 and 11.00h to collect brain samples. The experiment was performed in a balanced design in such a way that the control and noise-exposed rats were killed alternately to avoid the order effect. Brain samples were excised very quickly from the cranial cavity within 30sec after decapitation. The fresh brains were dipped in chilled saline (0.9% w/v); the hippocampus was isolated and stored at low temperature (−70°C) until analysis of 5-HT, 5-HIAA, DA, DOPAC, and HVA by HPLC-EC. HPLC-EC determination was carried out as the standard [30]. A 5-II Shim-Pack ODS separation column of 4.0mm internal diameter and 150mm length was used. Separation was achieved by a mobile phase containing methanol (14%), octyl sodium sulfate (0.023%), and EDTA (0.0035%) in a 0.1M phosphate buffer of pH 2.9 at an operating pressure of 2000–3000psi on the Schimadzu HPLC pump. Electrochemical detection was achieved on the Schimadzu LEC 6A detector at an operating potential of 0.8 volts.

### Cognitive Assessment

#### Novel Object Recognition Test

The experimental apparatus used for the object recognition task was an open field box (40×40×40cm) made of gray painted wood. The floor was covered with saw dust. The method was essentially the same as that described by Okuda et al. [31] with slight modification. The objects to be discriminated were two similar transparent glasses filled with white cement (A1 and A2) and a metallic container of the same size filled with white cement (new object, B). The test was comprised of three phases: the 1) Habituation phase 2) Training phase and 3) Test phase. On the 1st day, each rat was initially habituated to the open field box without any object for 15 minutes. On the 2nd day, each rat was placed in the open field for 15 minutes and allowed to freely explore two identical objects A1 and A2 (two glasses filled with white cement). On the 3rd day, during the test phase one, the object used during the training session was replaced by a novel object (B) and animals were left to explore the objects until they had accumulated 30 s of total object exploration time or for a maximum of 20 min. All objects presented similar textures, colors, and sizes, but distinctive shapes. A recognition index was calculated by the ratio T_B_/(T_A_ + T_B_). [T_A_ = time spent exploring the familiar object A; T_B_ = time spent exploring the novel object B] as described by de Lima et al. [32]. Exploration of an object was defined as sniffing or touching the object with the nose and/or forepaws. Turning around or sitting on the object was not considered as exploration.

#### Elevated Plus Maze Test

The Plus Maze Test is used to assess anxiety-like behavior in laboratory animals. The test has also been used to assess memory retention [33, 34]. The apparatus used in the present study consisted of two open arms (50×10 cm) crossed with two closed arms of the same dimensions with walls 40 cm high. The arms were connected with a central square (5×5 cm) to give the apparatus a plus sign appearance. The maze was elevated 60cm above the floor. To assess memory through the Eevated Plus Maze, the experimental session was comprised of two trials (1st day:Learning and 2nd day:Retention of memory). In the training session on the 1st day, rats were individually placed at one end of the open arm, facing away from the central platform, and the transfer latency (TL; time taken in seconds for the rat to move into one of the closed arms with all four paws) was recorded. The cut-off time during the training session was 2 minutes for the rat to explore the maze. The test session to evaluate the retention of memory was performed after 24 hours and the same procedure was repeated with a cut-off time of 60 sec. A significant increase in the transfer latency value of retention was taken as an index of impairment in memory.

### Statistical Analysis

The data were expressed as means ± SD. Data were analyzed by Student’s *t*-test and two-way ANOVA. Values P<0.05 were considered as significant.
